# The Fund Incorporated

**Published:** 1888-02-11

**Authors:** 


### THE NATIONAL PENSION FUND FOR NURSES.

### THE FUND INCORPORATED.

This fund has now been incorporated, the necessary documents having been signed on Friday and Saturday last. The signatories were: Lord Rothschild, Mr. Henry Hucks Gibbs (late Governor of the Bank of England), Mr. E. A. Hambro (Director of the Bank of England), and Mr. Junius S. Morgan, who worthily represent the merchant princes of the City of London; Mr. F. C. Carr Gomm (Chairman of the London Hospital) and Mr. Percival A. Nairne (Deputy Chairman of the Seamen's Hospital, Greenwich) represented the lay element; and Dr. J. S. Bristowe, F.R.S. (Senior Physician St. Thomas's Hospital), and Mr. Thomas Bryant, F.R.C.S. (Senior Surgeon of Guy's Hospital), the medical element in hospital administration. Mr. Burdett also signed as the Founder of the Fund. The first meeting of the Council has been summoned for Friday, the 10th inst., and arrangements are in progress which it is hoped will enable the prospectus to be ready not later than the 1st March next. The committees of the various institutions who are willing to co-operate, and all hospital officials and nurses who desire to join the Pension Fund, should send in their names without delay to the Acting Secretary, 38, Old Jewry, E.C., and so expedite the work of organisation.

